# Normotensive Palpitations in Primary Care Unmasking Pheochromocytoma Complicated by Reversible Cerebral Vasoconstriction Syndrome: A Case Report

**DOI:** 10.7759/cureus.100973

**Published:** 2026-01-07

**Authors:** Teresa Teixeira, Adriana Corte Real, Joana Silva, Jorge Teixeira, Samuel Canelas, Ana Azevedo

**Affiliations:** 1 Family Medicine, Unidade de Saúde Familiar (USF) Baguim-Unidade Local de Saúde (ULS) de Santo António, Gondomar, PRT; 2 Family Medicine, Unidade de Saúde Familiar (USF) São Bento-Unidade Local de Saúde (ULS) de Santo António, Gondomar, PRT

**Keywords:** adrenal pheochromocytoma, cortical subarachnoid hemorrhage (csah), palpitations, primary medical care, reversible cerebral vasoconstriction syndrome (rcvs)

## Abstract

Pheochromocytomas and paragangliomas (PPGLs) are rare catecholamine-secreting neuroendocrine tumors with intermittent and nonspecific manifestations that may be initially encountered in primary care. Although paroxysmal or sustained hypertension is a classic clue, some patients remain normotensive between episodes, which contributes to diagnostic delay. Reversible cerebral vasoconstriction syndrome (RCVS) is an important cause of thunderclap headache and may be complicated by cortical (convexity) subarachnoid hemorrhage (cSAH). Catecholamine excess is a recognized, treatable trigger of cerebral vasoconstriction and hemorrhagic/ischemic neurovascular events. We report a 50-year-old man followed in primary care for recurrent palpitations occurring 3-4 times per week with normal office blood pressure (BP) who later presented with thunderclap headache, and cSAH attributed to RCVS, with elevated BP at emergency presentation. Etiologic evaluation demonstrated markedly elevated plasma-free metanephrines and a hypervascular right adrenal mass consistent with pheochromocytoma. After guideline-concordant preoperative preparation with alpha-adrenergic blockade followed by beta-blockade, the patient underwent laparoscopic right adrenalectomy with a favorable perioperative course and biochemical remission on follow-up. This case highlights the pivotal role of primary care clinicians in the early recognition of PPGLs. Recurrent palpitations and other adrenergic spells should raise clinical suspicion and prompt diagnostic consideration even when hypertension is not documented. In a setting where primary care manages a large number of patients with established or newly diagnosed hypertension, timely biochemical testing and appropriate referral are essential to prevent severe, potentially life-threatening complications.

## Introduction

Pheochromocytomas are catecholamine-producing tumors arising from chromaffin cells of the adrenal medulla, while paragangliomas originate from extra-adrenal paraganglia; together they are termed PPGLs [1-3]. Although many patients present with the classic symptom cluster of episodic headache, palpitations, diaphoresis, and hypertension, the clinical phenotype is variable and may be intermittent, leading to misattribution to benign arrhythmia, anxiety/panic symptoms, thyroid disease, or primary headache disorders in ambulatory settings [1-3]. Contemporary recommendations emphasize biochemical testing with either plasma-free metanephrines or urinary fractionated metanephrines as first-line screening in patients with suggestive symptoms, adrenal incidentalomas, or relevant hereditary risk [4,5]. When PPGL is suspected, preoperative alpha-adrenergic blockade with volume repletion is essential to reduce perioperative hemodynamic instability; beta-blockade should only be added after adequate alpha-blockade (e.g., for persistent tachyarrhythmia) to avoid unopposed alpha-adrenergic stimulation [4-6].

Reversible cerebral vasoconstriction syndrome (RCVS) is characterized by sudden, severe (“thunderclap”) headaches and reversible multifocal cerebral arterial vasoconstriction. Complications include ischemic stroke, intracerebral hemorrhage, and convexity/cortical subarachnoid hemorrhage (cSAH) [7,8]. Identifying secondary triggers is clinically important because avoidance or definitive treatment of the precipitant may reduce recurrence and morbidity [7-9]. Catecholamine excess has been reported as a trigger for cerebral vasoconstriction and vasospasm with associated ischemic or hemorrhagic events, including in association with pheochromocytoma [10,11]. We present a case in which a pheochromocytoma was diagnosed after cSAH attributed to RCVS, highlighting practical learning points for earlier recognition in primary care, particularly when hypertension is not a prominent early feature.

## Case presentation

A 50-year-old man with a medical history of dyslipidemia and peripheral venous disease, previously treated with varicose vein surgery in 2007, without a contributory family history, was followed longitudinally in primary care. In March 2022, he first reported recurrent episodes of palpitations without associated alarm symptoms, prompting initial regular arrhythmia evaluation. Due to persistence of symptoms, propranolol 10 mg was initiated in April 2022 under the working diagnosis of benign palpitations/arrhythmia. Over the subsequent months, approximately 14 months (April 2022 to June 2023), he continued to experience paroxysmal episodes of tachycardia accompanied by pallor, diaphoresis, and self-limited abdominal discomfort, which did not initially prompt further diagnostic investigation. A temporary community supply shortage of propranolol later required a switch to nebivolol 1.25 mg for a few months.

In June 2023, after sudden exertion while running to catch the train, he developed an abrupt, severe headache consistent with a thunderclap presentation and sought emergency care. Neuroimaging demonstrated cSAH; aneurysmal etiology was excluded, and the overall clinical-radiologic picture supported RCVS. At the emergency presentation, elevated blood pressure (BP) was documented, and he required short intensive care monitoring with BP control. He was discharged with a target BP <140/90 mmHg and neurology follow-up.

**Figure 1:**
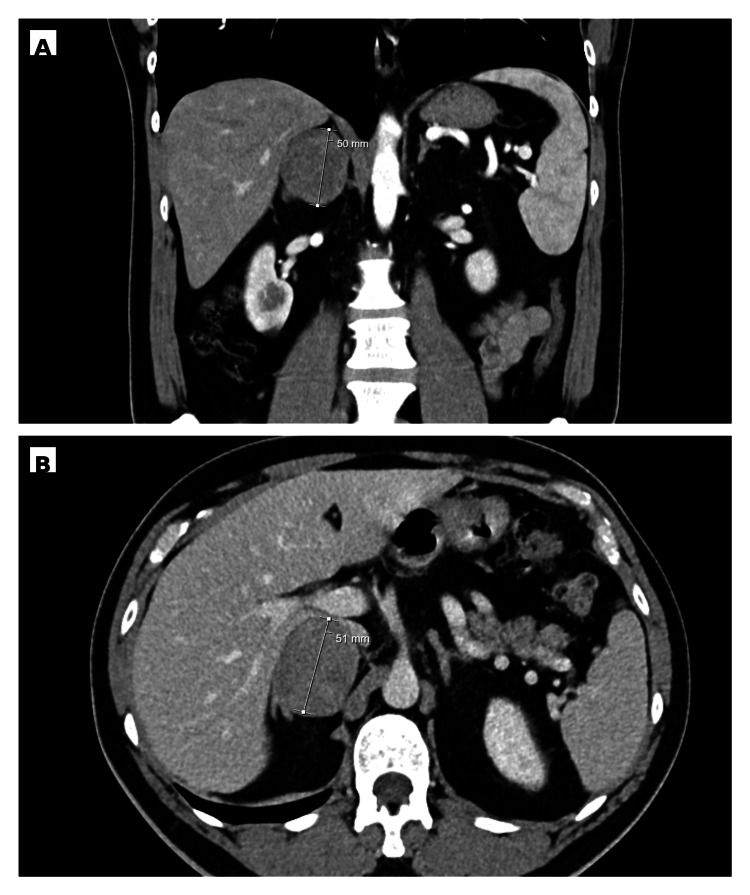
Contrast-enhanced abdominal CT demonstrating a hypervascular right adrenal mass (~5 cm) (A) Coronal reconstruction showing a right adrenal lesion with hypervascular enhancement and mass effect on adjacent structures. (B) Axial image confirming the right adrenal mass, with the maximum diameter measurement displayed

Given ongoing episodic adrenergic symptoms and BP variability after the neurovascular event, a targeted etiologic evaluation was pursued. Plasma-free metanephrines were markedly elevated (Table 1), and abdominal computed tomography identified a right adrenal hypervascular nodular lesion measuring approximately 4.4 × 5.2 × 4.5 cm, exerting mass effect without radiologic evidence of contiguous invasion (Figure 1). These findings supported the diagnosis of a catecholamine-secreting adrenal tumor consistent with pheochromocytoma; alternative adrenal or neurogenic lesions were considered, with definitive diagnosis established on histopathology.

**Table 1:** Plasma metanephrines

Analyte	Result (pmol/L)	Reference range (pmol/L)
Metanephrine	13,223	<456.3
Normetanephrine	6,088	<982.8
3-Methoxytyramine	123	<170

He was admitted electively in January 2024 for preoperative optimization with alpha-adrenergic blockade using phenoxybenzamine, followed by the addition of propranolol after adequate alpha-blockade had been established, in line with guideline principles. In March 2024, he underwent laparoscopic right adrenalectomy with an uncomplicated intra- and postoperative course and was discharged normotensive and asymptomatic.

Histopathology confirmed pheochromocytoma with a pheochromocytoma of the adrenal gland scaled score (PASS) of 0/20, indicating the absence of the adverse histologic features included in that scoring system. Postoperative follow-up demonstrated resolution of adrenergic symptoms and biochemical remission with normalization of metanephrines. Germline genetic testing (panel-based assessment) did not identify a pathogenic variant. At 12-month follow-up, he remained normotensive and asymptomatic, with complete resolution of palpitations and no recurrent adrenergic spells or further neurovascular events. Plasma metanephrines remained within the reference range, consistent with sustained biochemical remission. A shared-care surveillance plan was implemented, with annual endocrinology follow-up including repeat biochemical testing (plasma metanephrines, with additional catecholamine markers as clinically indicated) and clinical review, while primary care provided interim symptom assessment and BP monitoring twice yearly.

## Discussion

This case highlights several issues of direct relevance to primary care: (i) PPGLs may initially present with recurrent palpitations and adrenergic spells without sustained hypertension; (ii) catecholamine excess is a treatable precipitant of RCVS and cSAH; and (iii) early suspicion and appropriate testing in the community can reduce diagnostic delay and prevent major morbidity.

Why can PPGL be missed in primary care, especially without hypertension? Palpitations are among the most common reasons for ambulatory consultation and are frequently attributed to benign ectopy, anxiety, stimulant intake, or thyroid dysfunction. However, PPGL should remain on the differential when palpitations are recurrent, abrupt in onset, episodic, and accompanied by autonomic features (e.g., diaphoresis, pallor, tremor) or episodic headache, even if BP is normal at office visits [1-5]. This case illustrates that normal BP readings between episodes do not exclude PPGL; catecholamine secretion can be intermittent, and hypertension may be paroxysmal or emerge only during crises or complications [1-3,5]. In primary care, longitudinal follow-up is uniquely positioned to recognize patterns over time and to integrate seemingly isolated episodes into a coherent syndrome warranting biochemical screening.

RCVS/cSAH is a clue to secondary, treatable etiologies. RCVS is increasingly recognized as a leading cause of thunderclap headache. Hemorrhagic complications, including cSAH, occur in a clinically meaningful subset of patients and are associated with morbidity and mortality; therefore, identifying and treating triggers is central to management [7-9]. Catecholamine excess can provoke intense vasoconstriction, endothelial dysfunction, and BP surges, mechanisms plausibly linking pheochromocytoma to cerebral vasoconstriction and hemorrhagic phenomena [10,11]. While many RCVS cases are idiopathic or exposure-related, PPGL represents an upstream cause for which definitive treatment (surgical resection after proper blockade) can remove the precipitating stimulus and reduce future risk.

Beta-blockers and “symptom masking” in community practice

In primary care, beta-blockers are frequently prescribed to relieve palpitations, but in patients with unrecognized PPGL, they may attenuate adrenergic symptoms and inadvertently delay diagnosis. The patient initially received beta-blockade for palpitations, which is a common and often appropriate symptomatic strategy in primary care. Nevertheless, this case emphasizes two practical considerations. First, partial symptom control with beta-blockade can reduce the intensity of adrenergic symptoms and potentially delay recognition of the underlying catecholamine-secreting tumor. Second, when PPGL becomes a consideration, beta-blocker monotherapy should be avoided; guidelines recommend alpha-blockade first, with beta-blockade added only after adequate alpha-blockade to prevent unopposed alpha-adrenergic effects and hypertensive crisis [4-6]. In this patient, preoperative management followed this sequencing.

Pathology and follow-up implications

A PASS of 0/20 indicates that the tumor lacked the histologic features included in that system and is generally considered consistent with lower risk; however, PASS does not definitively predict behavior, and long-term recurrence can occur even after apparently complete resection [11]. Accordingly, structured follow-up with periodic biochemical testing is recommended, and primary care frequently plays a central role in ensuring adherence to surveillance and monitoring for recurrent symptoms [4,5].

## Conclusions

Pheochromocytoma is rare but curable, and delayed recognition can lead to catastrophic complications, including neurovascular events such as RCVS-associated cSAH. This case reinforces that primary care clinicians should consider PPGL in the differential diagnosis of recurrent palpitations and episodic adrenergic symptoms even when office BP is normal. Early biochemical testing with plasma-free or urinary fractionated metanephrines and timely referral enable safe preoperative preparation and definitive surgical treatment, reducing avoidable morbidity and mortality. Longitudinal surveillance after resection can be effectively coordinated in primary care to detect recurrence early.
